# Estimated Direct Medical Cost of Osteoporosis in Saudi Arabia: A Single-Center Retrospective Cost Analysis

**DOI:** 10.3390/ijerph18189831

**Published:** 2021-09-18

**Authors:** Bander Balkhi, Ahmed Alghamdi, Sulaiman Alqusair, Bader Alotaibi, Yazed AlRuthia, Hisham Alsanawi, Ahmad Bin Nasser, Mona A. Fouda

**Affiliations:** 1 Clinical Pharmacy Department, College of Pharmacy, King Saud University, Riyadh 11451, Saudi Arabia; aanahi@ksu.edu.sa (A.A.); yazeed@ksu.edu.sa (Y.A.); 2 College of Pharmacy, King Saud University, Riyadh 11451, Saudi Arabia; sulaiman.alqusair@gmail.com (S.A.); alotaibi.bader96@gmail.com (B.A.); 3 Department of Orthopedics, College of Medicine, King Saud University, Riyadh 11451, Saudi Arabia; halsanawi@ksu.edu.sa (H.A.); abinnasser@ksu.edu.sa (A.B.N.); 4 Department of Medicine, Endocrinology Division, College of Medicine, King Saud University, Riyadh 11461, Saudi Arabia; monafoudaneel@yahoo.com

**Keywords:** economic burden, costs, osteoporosis, Saudi Arabia

## Abstract

Osteoporosis and its complications are a major health concern in Saudi Arabia, and the prevalence of osteoporosis is on the rise. The aim of this study was to estimate the direct healthcare cost for patients with osteoporosis. A retrospective study was carried out among adult patients with osteoporosis in a teaching hospital in Saudi Arabia. A bottom-up approach was conducted to estimate the healthcare resources used and the total direct medical cost for the treatment of osteoporosis and related fractures. The study included 511 osteoporosis patients, 93% of whom were female. The average (SD) age was 68.5 years (10.2). The total mean direct medical costs for patients without fractures were USD 975.77 per person per year (PPPY), and for those with osteoporotic fractures, the total direct costs were USD 9716.26 PPPY, of which 56% of the costs were attributable to surgery procedures. Prior to fractures, the main cost components were medication, representing 61%, and physician visits, representing 18%. The findings of this study indicated the economic impact of osteoporosis and related fractures. With the aging population in Saudi Arabia, the burden of disease could increase significantly, which highlights the need for effective prevention strategies to minimize the economic burden of osteoporosis.

## 1. Introduction

Osteoporosis is defined as a skeletal disorder characterized by compromised bone strength predisposing a person to an increased risk of fracture. It is considered one of the most common diseases associated with aging, and it represents a serious public health concern in many developed countries [1]. The prevalence of osteoporosis continues to escalate with the increasingly elderly population, which poses a significant challenge to many healthcare systems having to deal with osteoporosis and other aging-related health conditions [2]. Globally, osteoporosis affects an estimated 200 million people [3]. In developed countries such as those in North America and Europe, as well as Japan and Australia, the country-specific osteoporosis prevalence ranges from 9% to 38% for women and from 1% to 8% for men [4]. The prevalence of osteoporosis is higher in women than in men; it is estimated that between 25% and 30% of all postmenopausal women in the United States and Europe have osteoporosis [5].

Osteoporosis is associated with a negative impact on several patient health domains such as physical, mental, and emotional wellbeing, with hip and vertebral fractures associated with the largest impact on patients’ quality of life [6]. The major complication of osteoporosis is an increase in fragility fractures, leading to significant morbidity, mortality, disability, and a decrease in patients’ quality of life [2,3]. Osteoporotic fracture can also lead to a loss of function, pain, and a decrease in daily activity, which can further lead to psychological issues such as anxiety and stress [7]. It has been estimated that at least one in three women older than 50 years and one in five men older than 50 years will experience a fragility fracture as a result of osteoporosis during their lifetime [8]. Globally, osteoporosis is responsible for more than 8.9 million fractures annually, of which 1.6 million are at the hip, 1.7 million are at the forearm, and 1.4 million are clinical vertebral fractures. Europe and the Americas account for 51% of all these fractures, while most of the remaining fractures occur in the Western Pacific and Southeast Asian regions [9]. By 2050, with the onset of population aging, the prevalence of osteoporotic hip fracture in Asia is projected to double, compared to the rates in 2018 [10]. 

In Saudi Arabia, accurate data on the current national incidence, prevalence, and associated treatments of osteoporosis are scarce. A study conducted among 1980 healthy adults in Jeddah estimated the prevalence rates of osteoporosis to be around 28.2% and 37.8% among women and men above 50 years of age, respectively [11]. Another study found that the prevalence rates of osteoporosis in women and men were 39.5% and 23.4%, respectively [12,13]. The overall, worldwide incidence of osteoporosis has been estimated at 4950/1,000,000 person–years [14].

The occurrence of osteoporosis continues to increase among older adults in Saudi Arabia despite the current advancements in healthcare systems, and there is an increase in the availability of proven effective medication to treat low bone mineral density and reduce the risk of fractures. Unfortunately, because osteoporosis is an asymptomatic disease, its management is still suboptimal, and this condition often remains undiagnosed and untreated until the advanced stage when fractures occur [15]. An analysis showed that more than 80% of Saudi patients who presented with a femoral fracture were never investigated and were treatment-naïve [14]. Another study from King Saud University Medical City (KSUMC) showed low screening and treatment rates for osteoporosis even after fragility hip fractures [16].

Through its prevalence and high healthcare resource requirements, osteoporosis is associated with significant health consequences for the individual and also puts a substantial economic burden on the individual, the government, and society, which is set to worsen in the future based on current population forecasting [11,17]. The average direct annual cost of osteoporosis in the U.S., Canada, and Europe was estimated to be between USD 5000 and USD 6500 billion [18]. In Saudi Arabia, the direct cost of osteoporosis-related femoral fractures was estimated at USD 150.60 million, while the total economic burden of osteoporosis-related fractures was estimated to be around USD 3.60 billion [14,19]. Osteoporosis-related hospitalization, followed by diagnosis testing and medications, was found to be the major driver of direct costs related to osteoporosis in Saudi Arabia [19]. 

As the Saudi population is growing, and because the number of elderly patients is expected to increase along with an increase in life expectancy, the incidence and prevalence of osteoporosis is expected to continue to increase [20]. In addition to the significant clinical burden osteoporosis imposes on individuals, the introduction of costly advances in health technology would add a considerable economic burden to healthcare systems in Saudi Arabia [21]. 

For action to be taken to reduce the burden, an accurate estimate of the cost attributed to osteoporosis in Saudi Arabia is critical to provide more insight into its economic burden; this will provide essential information to inform policies for the prevention and management of osteoporosis and to reduce associated costs.

The objective of this study is to estimate the direct medical cost of osteoporosis per patient in Saudi Arabia; to obtain a better understanding of the distribution of healthcare resources for this disease using real-world data for all patients, including those with and those without fractures, and to identify the main drivers of osteoporosis expenditure in the Saudi population, including subunit analysis by gender, which has not often been included in previous studies. The findings of this study will attempt to overcome the limitations of previous cost estimates that focused on fractures and lacked optimal estimates based on healthcare resources. Using a micro, ‘bottom-up’ costing approach, this study seeks to determine a more accurate estimation of the costs and healthcare resources associated with the management of osteoporosis.

## 2. Materials and Methods

### 2.1. Research Design

This was a single-center retrospective prevalence-based cost of illness (COI) study. It was conducted in KSUMC, the largest academic medical center in Riyadh that provides tertiary care to its patients. All patients with osteoporosis attending the center between January 2016 and December 2018 were identified and included. All healthcare resources used during the study period were quantified, and the total costs of osteoporosis treatment were estimated. In order to estimate the direct cost of resources used, a COI model was built via a bottom-up approach from electronic medical records. 

This approach was based on the costs of individual units of healthcare services performed. It uses the average cost of service estimates and applies these data to the total number of healthcare encounters related to the disease to arrive at the healthcare costs of a disease. This approach allows us to account for all related health resources and estimate the direct cost of osteoporosis-related fractures as a proxy of the total cost in Saudi Arabia.

### 2.2. Data Sources

This is a prevalence-based costing study using data from KSUMC. All fractures that were caused by osteoporosis were included in the study; diagnosis and treatment of fractures were identified based on the International Statistical Classification of Diseases and Related Health Problems ICD-10-AM (6th edition) and Systematized Nomenclature of Medicine diagnosis codes to ensure that all the patients were included at the time of hospital admission or emergency room visit. Estimations of the acute care costs, physician and prescription drug costs, and costs for rehabilitation, surgeries, hospital stay, and lab tests associated with osteoporosis were included.

### 2.3. Inclusion and Exclusion Criteria

#### 2.3.1. Inclusion

We included all osteoporotic patients, regardless of gender and type of fracture (hip, femoral, spine, wrist, or other), with a documented T score of <−2.5 from KSUMC. The total population that met the criteria comprised 511 osteoporotic patients.

#### 2.3.2. Exclusion

Patients with evidence of hip or vertebral fracture in the preceding year or with any other injury with multiple traumas were excluded, as were patients with fractures not related to osteoporosis and patients with other musculoskeletal disorders. Patients with pathologic fractures and patients with high-cost diseases such as cancer and chronic renal failure were excluded to prevent the overestimation of medical costs.

### 2.4. Cost Analysis

Cost analysis was performed from the perspective of the healthcare payer. All monetary values herein are expressed in U.S. dollars (USD) (1 USD = 3.75 Saudi Riyal). All costs were adjusted for inflation to 2020, using the Saudi Arabia Consumer Price Index. All the unit costs of the health resources were estimated using data from the KSUMC business center while the cost of medications was obtained from National Unified Procurement Company (NUPCO) which provided drugs with fixed tender price for all public hospitals. The direct cost encompassed the cost of all inputs for the management of patients with osteoporosis. Thus, the average direct medical costs for the whole duration of treatment and care were calculated and were further broken down by type of service (physician, inpatient hospitalization, procedure, outpatient, and rehabilitation) to identify the specific healthcare services responsible for the major costs within this period.

### 2.5. Data Analysis Plan

Descriptive statistics (mean, standard deviation, and frequency counts) were used to describe the characteristics of the patient population (age, sex, race, and geographic region). Continuous variables were summarized herein as mean cost; categorical data are summarized as absolutes and percentages. All statistical analyses were performed using IBM SPSS Statistics.

## 3. Results

The study included 511 osteoporosis patients, 93% of whom were female. The average (SD) age was 68.5 years (10.2). The baseline characteristics of all patients are shown in Table 1. Fractures occurred in only 57 (11%) of the patients. The most commonly occurring fractures were in the spine (40%) and femur (19%), followed by hip fractures (9%). 

The total direct medical costs for all osteoporotic patients included in this study were USD 996,824.29. Patients older than 65 years accounted for 72% of the total costs. The average annual cost per patient was USD 1950.73. The average cost for patients older than 65 years was higher than that for patients younger than 65 years: USD 2235.23 vs. USD 1470.08, respectively. The costs for male and female patients were USD 1961.95 and USD 1949.91, respectively (Table 2). Moreover, the average cost per person per year (PPPY) for non-fracture patients was USD 975.77, while the average cost PPPY for patients with osteoporotic fractures was USD 9716.26 PPPY and ranged from USD 4216.31 for patients who did not need surgery, to USD 31,249.16 for patients undergoing surgery. 

The major cost component for patients with osteoporosis-related fractures was surgery, which accounted for 56% of the total costs, followed by physiotherapy (20%). Before any fractures, the main cost components were medication (61% of costs), outpatient visits (18%), and diagnostic imaging (14%). The least contributing factor in the costs was lab tests, which accounted for only 3% of the total costs (Table 3). Diagnostic imaging accounted for around 8% of the total costs; the most commonly used methods were DXA (68%) and X-ray (28%). 

**Figure 1:**
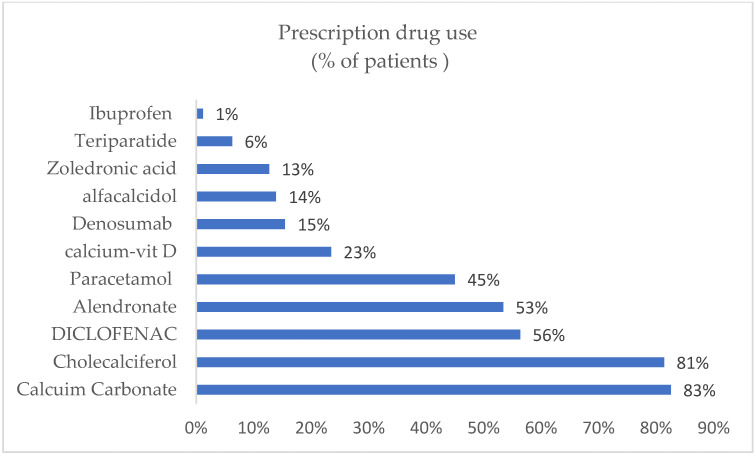
Medication utilization rates among patients with osteoporosis during the study period.

Figure 1 presents the utilization rates of medication used by the patients in this study. Calcium carbonate and cholecalciferol were used by 83% and 81% of patients, respectively. Diclofenac was used by 56% and alendronate was used by 53%. Overall, the estimated cost of medication used to treat patients with osteoporosis was USD 304,521.89, accounting for 31% of total costs. Although few patients in this study were treated with teriparatide, it accounted for almost 58% of the total medication costs, followed by denosumab at 12% of the total costs. On the other hand, analgesic treatment accounted for 9% of the total costs, and calcium and vitamin D treatment accounted for 8% of the total costs (Table 4).

## 4. Discussion

Osteoporosis and its associated complications have become a major public health concern in many countries, including Saudi Arabia. The growing prevalence of the disease, in addition to its substantial impact on morbidity, mortality, and the utilization of healthcare resources, especially among patients with fractures, has led to a significant economic burden on healthcare systems and societies [22]. Therefore, osteoporosis is considered a priority for health decision makers, due to the scarcity of healthcare resources and the need to prioritize healthcare policies and efficiently allocate the available health resources to control the growing economic burden of the disease. 

In this study, we performed a COI analysis, which was considered an important tool to support the setting of priorities in the healthcare decision-making process and to help in planning and predicting the current and future healthcare burden of osteoporosis. In this COI study, we adapted a payer perspective using real-world data and a bottom-up costing approach to estimate resource utilization, which provided a more accurate and detailed estimation of the direct medical costs associated with osteoporosis management. To the best of our knowledge, this is the first study in Saudi Arabia to provide a detailed breakdown and distribution of the costs regarding osteoporosis patients, with or without fractures, using real-world data. 

Our study showed that the total direct medical cost associated with the management of 511 patients with osteoporosis was USD 996,824.29, which indicated that the costs directly associated with osteoporosis management in Saudi Arabia were considerably high. The majority of the costs were related to the management of elderly female patients aged over 65 years, which was consistent with previous studies conducted in several countries, including the U.S., Taiwan, and Australia, where the cost of managing osteoporosis was found to be higher in patients of advanced age [23,24,25].

We also found that the annual costs per male patient are comparable to and slightly higher than the annual cost per female patient (USD 1961.95 vs. USD 1949.91, respectively). Although osteoporosis is more common among women in Saudi Arabia, detection of the disease is overlooked in men. Generally, osteoporosis is underdiagnosed or undertreated in men, which, in turn, is associated with a higher economic burden due to the fact that men tend to have severe osteoporosis-related fractures in advanced age, in addition to the presence of several comorbidities that increase the risk of fracture [26]. Our findings are similar to the reported results from previous studies in other countries and highlight the need for the proper detection and early management of osteoporosis in men to avoid possible delays and to consequently improve cost savings [27,28].

The findings of this study showed that 56% of the total direct costs were related to the management of osteoporosis-related fractures, which were reported in only 10% of the study population. Furthermore, the average PPPY cost for managing patients with fractures was USD 9716.26, approximately 10 times the cost of treating those without fractures (USD 975.77). A similar trend was reported in the U.S., and this demonstrated the significance of osteoporosis-related fractures as a major driver of osteoporosis expenditures, as compared to non-fracture-related costs [23]. Moreover, this estimate was higher than those from other countries, such as USD 3119 in Turkey, USD 3800 in Portugal, USD 5545 in Singapore, and USD 7860 in China. In contrast, our estimate was lower than the estimated cost reported in the U.S. (USD 19,225) [18].

This variation in cost estimates reported from different countries may be due to the methodology used for estimating costs, treatment protocol, and prices of healthcare resources. However, it also indicates the importance of using local data to estimate the COI and to produce an accurate representation of the actual cost to properly inform decision making.

Among patients with fractures, our study revealed that the PPPY cost could reach up to USD 31,249.16 for patients undergoing surgeries, including hip replacement, and fractures largely drove the costs of procedures, physiotherapy, and hospitalization. This finding is astronomically higher than the costs reported by previous local studies that ranged from USD 12,868 to USD 20,000 [14,19]. This indicates that the economic burden associated with osteoporosis-related fractures is significant and was previously underestimated. The substantial increase in our study could be attributed to the inclusion of more cost categories and resources consumed by patients with osteoporosis, in addition to the usage of data on the actual costs of healthcare resources, which have substantially increased over the last few years, unlike previous studies that relied mainly on external or outdated local costing data. 

The results of our study showed the distribution across all direct cost categories of patients with fractures and without. Costs attributed to surgeries and hospitalization were the major driver of expenditures among patients with fractures, followed by physiotherapy costs, accounting for 70% and 20%, respectively. In contrast, among patients without fractures, medication costs were the major driver of expenditures, accounting for 61%, followed by costs for outpatient visits, accounting for 18%. Similar findings were reported in countries such as Canada, where surgeries and hospitalizations accounted for 76% of the costs associated with the management of patients with osteoporosis with fractures, whereas medications and outpatient visits accounted for 69% and 20%, respectively, of the management costs of osteoporosis without fractures [29].

Our study indicates the importance of planning an effective prevention strategy to minimize the total expenditure related to fractures. The cost data associated with fractures in our study could be beneficial for assessing the value of any future policies that could prevent osteoporosis-related fractures. Despite the adaption of several initiatives by the Ministry of Health in Saudi Arabia, there are still unmet needs and gaps in the management and prevention of osteoporosis and related complications, including fractures, all of which might lead to a potential economic impact on the healthcare system. These gaps have resulted in lower rates of screening, a larger number of patients being either undiagnosed or not treated properly before fractures, limited post-fracture care, and reliance on specialists as major providers for healthcare and follow-up services, rather than primary care practitioners [30,31]. Hence, our study confirms the importance of implementing cost-effective and cost-saving screening, treatment, and services to reduce the growing expenditures associated with fragility fractures.

One approach that might be adapted by the Saudi healthcare system is evaluating the cost effectiveness of the universal screening program for fractures in patients with osteoporosis who are at high risk and those of advanced age, supported by improving public awareness and improving healthcare infrastructure training for healthcare professionals. Several strategies of screening in both genders—including screening with DXA and pre-screening with FRAX, followed by drug therapy—would be cost-effective and would improve cost savings and quality of life compared to no screenings [32,33,34]. Furthermore, another approach that is worth further investigation in Saudi Arabia is adapting secondary preventive strategies after fractures, including fracture liaison services. The Fracture Liaison Service centers that have been started in Saudi Arabia, including KSUMC, are on the international map for best practice in the “capture the fracture” initiative by the International Osteoporosis Federation [17,35]. This approach has proven to be cost-effective and even cost-saving, in addition to effectively lowering future fracture rates by almost 80% [36,37,38].

Drug utilization across the entire study population revealed that calcium and vitamin D supplements were utilized the most, followed by analgesics and bisphosphonates. Although the consumption of the drugs denosumab and teriparatide was low, both drugs contributed to approximately 70% of total drug costs. These two drugs were effective and usually restricted to certain groups, including those with high risk of fracture. However, both agents were expensive, and their added value compared to standard treatments, including bisphosphonates, varied across countries [39]. Hence, it is critical for decision makers to properly evaluate the expected added value and affordability for the healthcare system, given the limited economic evidence and the increasing number of patients who are at risk of fracture and need access to these expensive drugs in Saudi Arabia. Costs related to diagnostic and lab tests were not substantially high in this study, due to lower rates of fracture assessments (only 7% of the population) and lower utilization rates for osteoporosis-specific diagnostic and lab tests, such as DXA scans, bone profiles, and vitamin D and thyroid function tests. Other secondary causes for screening included CBC for anemia, renal function and liver function tests, malabsorption tests such as for celiac disease, and specific hormonal tests when indicated.

### Limitations

This study provides further insights on the economic burden of osteoporosis in Saudi Arabia from a payer perspective relying on real-world costing data. However, there are some important limitations that should be noted. First, the estimated fracture costs mainly focused on those related to spine and hip fractures and did not account for other types of fractures, due to the limited number of patients. Even though the finding of this COI study is considered representative for the government in general, it may not be generalizable to private institutions, as the cost components between healthcare services could differ dramatically. In addition, our study only included a direct medical cost estimation and did not account for other types of costs, such as direct non-medical or indirect costs, which could have a potential impact on the economic burden of osteoporosis. The indirect cost of osteoporosis-related fractures was estimated to be approximately USD 451.7 million in 2015 in Saudi Arabia [14]. However, this estimate is outdated and may underestimate the actual costs, due to limitations in the methodology used to estimate the cost. Future studies should consider the actual impact of indirect costs and direct non-medical costs on the overall economic burden in Saudi Arabia.

## 5. Conclusions

Osteoporosis and its related fractures impose a large economic burden on patients in Saudi Arabia. With the growing elderly population in Saudi Arabia, the burden of disease could increase significantly, highlighting the importance of initiating effective prevention strategies, including promoting bone health, implementing early diagnosis programs, and increasing access to effective treatment in order to minimize the economic burden of osteoporosis and reduce the costs incurred by the health system. Current information about the size of the financial burden of osteoporosis, included in our study, will create awareness about this issue. It may also help guide future policies and research toward evaluating both the clinical and economic benefits of new health technologies and surgery options, in order to allocate resources more efficiently.

## Figures and Tables

**Table 1:** Characteristics of patients with osteoporosis included in the study.

Variables	N (%)
Age in years, mean (SD)	69 (10.26)
Age group in years, *n* (%)	
<65	190 (37)
>65	321 (63)
Gender, *n* (%)	
Female	476 (93)
Male	35 (7)
Fracture type, *n* (%)	
Spine	23 (40)
Femur	11 (19)
Hip	6 (11)
Metatarsal fractures	5 (9)
Wrist	4 (7)
Humerus	3 (5)
Ankle	2 (4)
Rib	2 (4)
Pelvis	1 (2)
No. of fractures, *n* (%)	
1	48 (84)
2	7 (12)
3	1 (2)
4	1 (2)

**Table 2:** Osteoporosis-related costs based on gender and age group.

Variables	Total Cost	Cost Per Patient	% of Total Cost
Sex			
Male	USD 68,668.44	USD 1961.95	7%
Female	USD 928,155.85	USD 1949.91	93%
Age			
<65	USD 279,315.91	USD 1470.08	28%
>65	USD 717,508.37	USD 2235.23	72%
Fractures			
Yes	USD 553,826.94	USD 9716.26	56%
No	USD 442,997.34	USD 975.77	44%

Note: costs are in U.S. dollars.

**Table 3:** Cost estimation for patients with osteoporosis.

		Annual Cost Per Patient (USD )			
**Cost Items**	**Total Cost (USD )**	**Non Fracture**	**%**	**Fracture**	**%**
Lab test	USD 33,952.00	USD 61.60	6%	USD 94.13	1%
Diagnostic test	USD 75,203.73	USD 141.33	14%	USD 173.33	2%
Hospitalization	USD 80,000.00	-	0%	USD 1356.00	14%
Visit	USD 90,880.00	USD 177.07	18%	USD 160.00	2%
Physiotherapy	USD 106,400.00	-	0%	USD 1952.29	20%
Surgery	USD 305,866.67	-	0%	USD 5461.90	56%
Medication	USD 304,521.89	USD 595.77	61%	USD 518.66	5%
Total	USD 996,824.29	USD 975.77		USD 9716.26	

Note: costs are in U.S. dollars.

**Table 4:** Costs of medications for the treatment of osteoporosis during the study period.

Drug	Annual Treatment Cost	% of Total Cost
Alendronate	USD 27,138.37	8.9%
Zoledronic acid	USD 6980.13	2.3%
Denosumab	USD 37,653.71	12.4%
Teriparatide	USD 177,029.12	58.1%
Paracetamol	USD 12,806.40	4.2%
Cholecalciferol	USD 17,410.99	5.7%
Calcium carbonate	USD 2433.33	0.8%
Diclofenac	USD 16,404.48	5.4%
Alfacalcidol	USD 1382.13	0.5%
Calcium–Vitamin D	USD 4438.40	1.5%
Ibuprofen	USD 844.80	0.3%
Total cost	USD 304,521.87	100.0%

## Data Availability

The original contributions presented in the study are included in the article; further inquiries can be directed to the corresponding author.
